# Physiological Responses to Hypoxia on Triglyceride Levels

**DOI:** 10.3389/fphys.2021.730935

**Published:** 2021-08-23

**Authors:** Renée Morin, Nicholas Goulet, Jean-François Mauger, Pascal Imbeault

**Affiliations:** ^1^School of Human Kinetics, Faculty of Health Sciences, University of Ottawa, Ottawa, ON, Canada; ^2^Hôpital Montfort, Institut du Savoir Montfort, Ottawa, ON, Canada

**Keywords:** intermittent hypoxia, continuous hypoxia, dyslipidemia, triglycerides, triglyceride-rich lipoproteins

## Abstract

Hypoxia is a condition during which the body or specific tissues are deprived of oxygen. This phenomenon can occur in response to exposure to hypoxic environmental conditions such as high-altitude, or because of pathophysiological conditions such as obstructive sleep apnea. Circumstances such as these can restrict supply or increase consumption of oxygen, leading to oxyhemoglobin desaturation and tissue hypoxia. In certain cases, hypoxia may lead to severe health consequences such as an increased risk of developing cardiovascular diseases and type 2 diabetes. A potential explanation for the link between hypoxia and an increased risk of developing cardiovascular diseases lies in the disturbing effect of hypoxia on circulating blood lipids, specifically its capacity to increase plasma triglyceride concentrations. Increased circulating triglyceride levels result from the production of triglyceride-rich lipoproteins, such as very-low-density lipoproteins and chylomicrons, exceeding their clearance rate. Considerable research in murine models reports that hypoxia may have detrimental effects on several aspects of triglyceride metabolism. However, in humans, the mechanisms underlying the disturbing effect of hypoxia on triglyceride levels remain unclear. In this mini-review, we outline the available evidence on the physiological responses to hypoxia and their impact on circulating triglyceride levels. We also discuss mechanisms by which hypoxia affects various organs involved in the metabolism of triglyceride-rich lipoproteins. This information will benefit scientists and clinicians interested in the mechanistic of the regulatory cascade responsible for the response to hypoxia and how this response could lead to a deteriorated lipid profile and an increased risk of developing hypoxia-related health consequences.

## Introduction

Through evolution, organisms have developed physiological systems to maintain and regulate oxygen (O_2_) homeostasis (Semenza, 2000). O_2_ plays the most important role in vegetal and animal respiration serving as the electron acceptor during oxidative phosphorylation (Brahimi-Horn and Pouysségur, 2007). Hypoxia is a condition during which the body is deprived of adequate O_2_ at the tissue level. Essentially, hypoxia occurs when O_2_ demand is greater than O_2_ delivery, initiating changes in gene expression mediated by a class of transcriptional factors called hypoxia-inducible factors (HIF). HIF-1, one of 3 major HIF isoforms, is known as the master regulator of cellular responses to hypoxia and activates the transcription of more than 1,000 genes encoding enzymes, as well as transport and mitochondrial proteins controlling the delivery and utilization of O_2_ (Semenza, 2014). Environmental conditions such as high altitude, or pathological conditions such as obstructive sleep apnea (OSA) and chronic obstructive pulmonary disease can lead to tissue hypoxia (Raguso et al., 2004; Drager et al., 2010). In some conditions (e.g., OSA), individuals exposed to hypoxia are at an increased risk of developing cardiovascular diseases (CVD) and metabolic disorders (Drager et al., 2013). A potential explanation linking hypoxia to these health consequences lies in its disturbing effect on lipid metabolism. More precisely, hypoxia may disturb the balance between lipid storage and lipid mobilization in hepatic and adipose tissues (AT). These disturbances, characterized by an overproduction and/or an impaired clearance of triglyceride-rich lipoproteins (TRL), can lead to a deterioration of the blood lipid profile, specifically an increase in plasma triglyceride (TG) concentrations. Prolonged impairment of lipid storage and/or mobilization and overexposure of non-adipose tissue to high plasma lipid concentrations can in the long-term lead to lipotoxicity, which could promote the development of metabolic disorders (Lewis et al., 2002). Consequently, considerable research has investigated the effect of acute or chronic hypoxia on circulating TG levels. The following sections will provide key information on TG metabolism, experimental evidence linking hypoxia and circulating TG levels, and the main underlying mechanisms by which hypoxia modulates TG levels.

## Importance, Production, and Effects of Triglycerides

TG are the most energy-dense substrate. They can be derived from food or produced through the conversion of excess carbohydrates and amino acids into fatty acids, through a process called *de novo* lipogenesis, that are then esterified to glycerol to produce TG. TG are composed of 3 fatty acids and 1 glycerol, and the long aliphatic chains provided by the fatty acids confers a highly hydrophobic character to the molecule. The transportation of TG in the aqueous environment typical of living organisms thus requires the help of specialized proteins called lipoproteins. Blood TG are secreted in the circulation in the form of TRL by the liver [as very-low-density lipoproteins (VLDL)] and the intestine [as chylomicrons (CM)] (Chapman et al., 2011; Packard et al., 2020). Humans ingest approximately 30–150 g of TG per day (Dubois et al., 1998). The small intestine’s main roles include the digestion, absorption, and finally the secretion of dietary fats into the circulation in the form of CM (Xiao et al., 2011). In humans, a minimal consumption of 15 g of lipids is needed to induce a significant increase in postprandial CM levels (Dubois et al., 1998). Peak TG levels typically occur 2–4 h following meal ingestion. In the fasted state, normal TG levels should be lower than 1.7 mmol/L; higher values represent a CVD risk factor (Miller et al., 2011). It should also be noted that postprandial TG levels are superior to fasting TG as predictors of CVD risk (Kolovou and Ooi, 2013). Elevated triglyceridemia, both in fasting and postprandial states, is therefore increasingly considered a risk factor for CVD (Kolovou and Ooi, 2013).

## Triglyceride Metabolism

**FIGURE 1:**
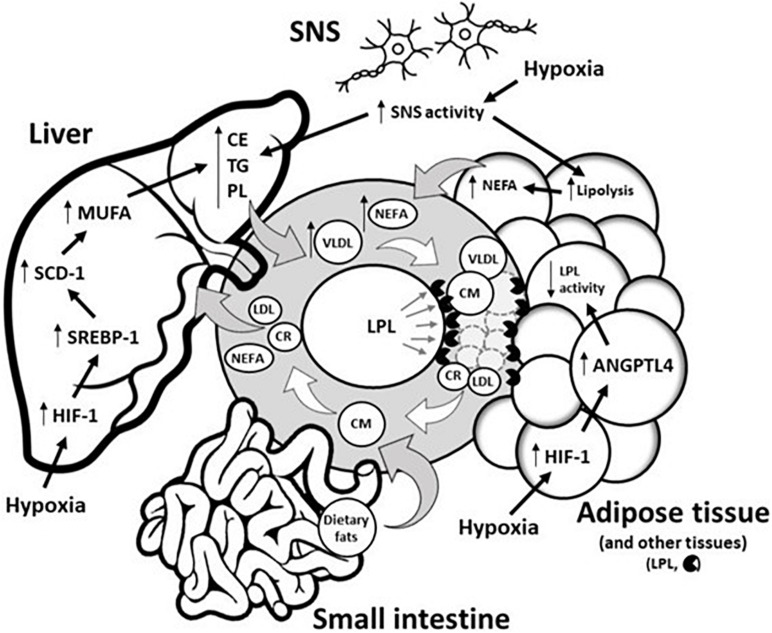
Potential underlying physiological mechanisms by which hypoxia modulates blood triglyceride levels. Circulating TG levels reflect the production of TRL by both the liver (as VLDL) and the intestine (as CM) as well as the disposal of TRL and their remnants (LDL and CR) in the liver/periphery. Hypoxia does not disturb CM metabolism in the small intestine. Conversely, hypoxia disturbs hepatic and adipose tissue functions by (1) delaying hepatic removal of TRL remnants from circulation, and/or (2) delaying intravascular lipolysis of TRL via the HIF-1 upregulation of ANGTPL4, a potent inhibitor of adipose tissue LPL activity. In the liver, hypoxia also increases lipid biosynthesis via HIF-1-dependent upregulation of the transcriptional factor SREBP-1, which increases expression of SCD-1, the rate-limiting enzyme for the synthesis of MUFA, a major substrate for the synthesis of CE, TG, and PL. These latter substrates are key determinants involved in VLDL production. Through sympathetic activation, hypoxia can also increase VLDL production by (1) directly increasing VLDL assembly in the liver, and/or (2) by stimulating intracellular lipolysis of adipose tissue, which results in the increased release of NEFA, the main precursor to VLDL production. ANGPTL4, Angiopoietin-like 4; CM, Chylomicron; CR, Chylomicron remnant; CE, Cholesterol ester; HIF-1, Hypoxia-inducible factor 1; LDL, Low-density lipoprotein; LPL, Lipoprotein lipase; MUFA, Monounsaturated fatty acid; NEFA, Non-esterified fatty acid; PL, Phospholipid; SCD-1, Stearoyl-CoA desaturase-1; SREBP-1, Sterol regulatory element-binding protein 1; SNS, Sympathetic nervous system; TG, Triglyceride; VLDL, Very-low-density lipoprotein.

Circulating TG levels reflect the balance between the production of TRL by the liver (as VLDL) and the intestine (as CM), and the disposal of TRL and their remnants by the periphery and the liver (Chapman et al., 2011; Packard et al., 2020; Figure 1). Following the ingestion of at least 15 g of lipids, a transient increase in TG levels and a change in plasma lipoprotein pattern occur (Lopez-Miranda et al., 2007). In brief, both the plasma concentrations of CM of intestinal origin and VLDL remnants increase (Nakajima et al., 2011). In contrast, in the postabsorptive state, TG are predominantly produced by the liver as VLDL. Both TRL are mainly hydrolyzed by the endothelium-bound enzyme lipoprotein lipase (LPL) which is secreted by AT and other tissues. Following the hydrolysis of VLDL and CM, low-density lipoprotein and CM remnants are metabolized by the liver to resynthesize VLDL (Figure 1). The secretion of VLDL by the liver is modulated according to the hormonal/nutritional state. During postabsorptive status or short-term fasting, the activation of the sympathetic nervous system (SNS) and the secretion of stress hormones facilitate the intracellular breakdown of TG within AT, which favor the efflux of non-esterified fatty acids (NEFA) into the circulation (Lafontan and Langin, 2009; Young and Zechner, 2013). NEFA are the main substrate for VLDL production (Xiao et al., 2011). Conversely, in postprandial or prandial states, insulin is secreted by the pancreas and strongly suppresses AT intracellular lipolysis, resulting in a reduced hepatic NEFA influx which represses hepatic VLDL production (Xiao et al., 2011; Nielsen and Karpe, 2012). Other non-negligible contributors to hepatic VLDL production are the *de novo* lipogenesis pathway and the uptake of TRL remnants. In summary, hepatic VLDL synthesis is mainly substrate-driven and primarily determined by hepatic lipid availability, to which NEFA delivery appears to be the main contributor (Nielsen and Karpe, 2012).

## Experimental Evidence Linking Hypoxia and Circulating Triglyceride Levels

The following section discusses the results found in Table 1. For over half a century, hypoxia has been associated with lipemic disturbances (Louhija, 1969). Table 1 summarizes experimental evidence from rodent and human studies reporting the effects of acute/chronic and intermittent/continuous hypoxia on AT-LPL activity and on circulating TG and NEFA concentrations. Studies were included if they simulated hypoxia in a controlled environment and/or had a weight-matched control group if the exposition time was longer than 1 day. Hypoxia is consistently associated with elevated TG levels in murine models, with 12 out of 14 studies reporting an increase ranging from 20 to 570%, and no studies reporting a decrease. This wide range of increased TG levels could stem from several factors, including the type and severity of hypoxic exposure, diet, exposition time, nutritional status, temperature, and inter-species differences. Most notably, a negative relationship between the fraction of inspired oxygen (FiO_2_) and TG levels has been observed (Jun et al., 2012).

**TABLE 1:** Effects of acute/chronic and intermittent/continuous hypoxia on adipose tissue LPL activity, and on circulating levels of triglycerides and non-esterified fatty acids in rodent and human studies.

References	Year	IH/CH	Exposition time	Hypoxic severity	Adipose tissue LPL activity	Blood triglycerides	Blood non-esterified fatty acids	Nutritional status	Rodent/Human
Louhija (1969)	1969	CH	12 days	FiO_2_ = 0.10	N/A	↑ 570%	No differences	Fasting	Sprague-Dawley rats
Férézou et al. (1993)	1993	CH	7 days	FiO_2_ = 0.12	N/A	No differences	N/A	Fasting	Healthy young participants
					N/A	↓ 40%	N/A	Postprandial	Healthy young participants
Leaf and Kleinman (1996)	1996	CH	2 h	FiO_2_ = 0.16	N/A	No differences	N/A	Fasting	Older men
Muratsubaki et al. (2003)	2003	CH	5 h	FiO_2_ = 0.10	N/A	↑ 210%	N/A	Sated	Sprague-Dawley rats
					N/A	No differences	N/A	Fasting	Sprague-Dawley rats
Li et al. (2005)	2005	IH	5 days	60 cycles/h FiO_2_ = 0.05	N/A	↑ 40%	No differences	Fasting	C57bl/6j mice, not thermoneutral, lean
					N/A	No differences	No differences	Fasting	C57bl/6j mice, not thermoneutral, obese
Li et al. (2007a)	2007	IH	4 weeks	FiO_2_ = 0.05	N/A	↑ 25%	N/A	Fasting	C57bl/6j mice, not thermoneutral,
				FiO_2_ = 0.10	N/A	No differences	N/A	Fasting	C57bl/6j mice, not thermoneutral,
Li et al. (2007b)	2007	IH	4 weeks	60 cycles/h FiO_2_ = 0.05	N/A	↑ 100%	No differences	Fasting	Scap*fl/fl* mice, not thermoneutral
					N/A	No differences	No differences	Fasting	L-Scap- mice, not thermoneutral, deficiency in SREBP
Savransky et al. (2007)	2007	IH	12 weeks	60 cycles/h FiO_2_ = 0.05	N/A	No differences	N/A	Fasting	C57bl/6j mice, not thermoneutral, regular diet
					N/A	No differences	N/A	Fasting	C57bl/6j mice, not thermoneutral, high cholesterol diet
Savransky et al. (2008)	2008	IH	10 weeks	60 cycles/h FiO_2_ = 0.05	N/A	No differences	No differences	Fasting	C57bl/6j mice, not thermoneutral
					N/A	No differences	No differences	Fasting	C57bl/6j mice, not thermoneutral, treated with antisense SCD-1 oligonucleotides
Jun et al. (2010)	2010	IH	4 weeks	60 cycles/h FiO_2_ = 0.07	N/A	↑ 60%	↑ 45%	Fasting	ApoE-/- mice, not thermoneutral
Drager et al. (2012)	2012	IH	4 weeks	60 cycles/h FiO_2_ = 0.07	↓ 80%	↑ 100%	↓ 15% *	Fasting	C57bl/6j mice, not thermoneutral
Jun et al. (2012)	2012	CH	6 h	FiO_2_ = 0.07	↓ 60%	↑ 150%	↑ 60%	Fasting	C57bl/6j mice, not thermoneutral
Jun et al. (2013)	2013	CH	6 h	FiO_2_ = 0.10	↓ 60%	↑ 50%	↓ 40%	Fasting	C57bl/6j mice, not thermoneutral
					↓ 80%	No differences	No differences	Fasting	C57bl/6j mice, thermoneutral
Drager et al. (2013)	2013	IH	4 weeks	60 cycles/h FiO_2_ = 0.07	N/A	↑ 55%	↑ 30%	Fasting	ApoE-/- mice, not thermoneutral
					N/A	No differences	↑ 35%	Fasting	ApoE-/- mice, not thermoneutral, treated with ANGPTL4-neutralizing antibody
Yao et al. (2013)	2013	IH	4 weeks	60 cycles/h FiO_2_ = 0.07	↓ 50%	↑ 20%	N/A	Fasting	C57bl/6j mice, not thermoneutral
					No differences	↑ 75%	N/A	Fasting	C57bl/6j mice, not thermoneutral, treated with ANGTPL4-neutralizing antibody
Siques et al. (2014)	2014	CH	30 days	FiO_2_ = 0.12	N/A	↑ 35%	N/A	Fasting	Winstar rats, not thermoneutral
Mahat et al. (2016)	2016	IH	6 h	17 cycles/h N_2_ = 100%	No differences	No differences	↑ 25%	Postprandial	Healthy young men
Chopra et al. (2017)	2017	IH	3 nights	CPAP withdrawal	N/A	No differences	↑	Postprandial	OSA patients
Mahat et al. (2018)	2018	CH	6 h	FiO_2_ = 0.12	N/A	No differences	↑ 95%	Fasting	Healthy young men
Mauger et al. (2019)	2019	CH	6 h	FiO_2_ = 0.12	N/A	↑ 15% *	↑ 30%	Prandial	Healthy young men
Morin et al. (2021)	2021	IH	6 h	15 cycles/h N_2_ = 100%	N/A	↑ 45%	↑ 25% *	Postprandial	Healthy young men
					N/A	No differences	No difference	Postprandial	OSA patients

ANGPTL4, Angiopoietin-like 4; CH, continuous hypoxia; FiO_2_, fraction of inspired oxygen; IH, intermittent hypoxia; LPL, lipoprotein lipase; OSA, obstructive sleep apnea; SCD-1, Stearoyl-CoA desaturase-1; SREBP-1, Sterol regulatory element-binding protein 1. *Trend (p-value between 0.05 and 0.1).

A major concern regarding the physiological relevance of studies conducted with rodents is the effect of thermoneutrality. Jun et al. (2013) compared the effect of acute continuous hypoxia in mice exposed to either a thermoneutral condition (30°C) or a non-thermoneutral condition (22°C). They showed that cooler ambient temperatures increased lipid uptake in several tissues (e.g., liver, lungs, heart, brown, and white adipose tissues) and that hypoxia attenuated this stimulation, resulting in increased TG levels. Under the thermoneutral condition, no difference was observed in the lipid uptake of these tissues, and TG levels were not altered following 6 h of normoxia vs. hypoxia (FiO_2_ = 0.10) exposure. These findings led the authors to conclude that ambient temperatures must be carefully considered in the design and interpretation of experiments that involve hypoxia in mice. This statement likely applies to experiments with humans given the well-recognized effects of cooler temperature on energy metabolism (van Marken Lichtenbelt et al., 2002; Blondin et al., 2017). Throughout the last couple of decades, rodent studies have also identified other factors that can weaken the disturbing effect of hypoxia on lipid metabolism. Those factors include, but are not limited to, pre-existing obesity (Li et al., 2005) and deficiencies in certain nuclear factors/proteins (Li et al., 2007a; Savransky et al., 2008; Drager et al., 2013; Yao et al., 2013).

Human studies do not report elevated TG levels in response to hypoxia as consistently as in murine studies. Leaf and Kleinman (1996) observed no changes in fasting TG levels in individuals (mean age = 70 years) who underwent 2 h of continuous hypoxia exposure. More recently, Mahat et al. (2018) investigated the effect of a 6-h exposure to normobaric continuous hypoxia (FiO_2_ = 0.12) in fasted healthy young men and reported no difference in TG levels. The same group also investigated the effect of acute intermittent hypoxia (IH) on postprandial TG levels in healthy young men in 2 independent studies with conflicting results despite using similar IH protocols. In one study, no difference in postprandial TG levels between IH and normoxia exposure was observed (Mahat et al., 2016), while in the other, a marginal transient increase in postprandial TG levels was observed after 3 h of IH exposure (Morin et al., 2021). This discrepancy is not readily explainable, but we speculate that it could be the result of higher postprandial peaks in TG levels achieved in the latter study. A study conducted in a steady prandial state provided further insight into the effect of hypoxia on lipid metabolism by showing a trend toward 15% higher prandial TG levels after 4 h of normobaric continuous hypoxia (Mauger et al., 2019).

The effect of hypoxia on circulating lipids has also been examined in pathophysiological conditions such as OSA, a sleep disorder characterized by episodes of partial or complete obstruction of the upper respiratory airways that leads to IH during sleep (Dempsey et al., 2010). Chopra et al. (2017) compared individuals with OSA, untreated or treated using continuous positive airway pressure (CPAP) withdrawal. CPAP withdrawal increased nocturnal NEFA but not TG levels. In line with this observation, Morin et al. (2021) recently showed no difference in postprandial TG in individuals with OSA exposed to 6 h of IH or ambient air, in a crossover design.

In summary, there is a consistent increase in TG levels in response to hypoxia in rodents while such a response is less obvious in human studies (Table 1). It is critical to keep in mind that the effect of thermoneutrality on circulating TG levels may be an important concern regarding the physiological relevance of studies conducted with rodents. In humans, studies have always been conducted in thermoneutrality. Would conducting human trials in non-thermoneutral conditions lead to similar inter-species results? This warrants further exploring. In rodents, it has been shown that hypoxia increases TG levels through two major mechanisms: increased hepatic secretion of VLDL (Li et al., 2007a) and decreased TRL clearance (Jun et al., 2012; Drager et al., 2013). In human studies, cellular and molecular mechanisms are still poorly understood and require further attention. Despite progress and recent developments, several questions remain, including the effects of age, sex, as well as the exact hypoxic threshold (severity × time) needed to trigger modulations of TG levels. The sections below elaborate on the knowledge to date pertaining to the underlying mechanisms by which hypoxia modulates circulating TG levels.

## Underlying Mechanisms by Which Hypoxia Modulates Triglycerides Levels

### White Adipose Tissue

White AT has an important buffering action on circulating lipids concentrations (e.g., NEFA and TG contained in lipoproteins) by modulating (1) the release of NEFA into circulation, which occurs through intracellular lipolysis, and (2) the clearance of TRL-TG, which occurs through intravascular lipolysis of TRL’s content. The effects of hypoxia on these crucial pathways are discussed in this section.

#### Intracellular Lipolysis

In periods of food deprivation and/or excess metabolic need, TG stored in AT can be rapidly mobilized into the circulation as NEFA and glycerol. The catabolic process of intracellular lipolysis relies on 3 hydrolases, of which 2, namely the hormone-sensitive lipase (HSL) and the adipose TG lipase (ATGL), are considered regulated and rate-limiting (Lafontan and Langin, 2009; Young and Zechner, 2013). The HSL and ATGL are mainly under hormonal control with catecholamines and natriuretic peptides stimulating, and insulin inhibiting ATGL, HSL, and AT lipolysis. Hypoxia has been shown to increase SNS tone (Somers et al., 1989, 1995) and catecholamines secretion (Mesarwi et al., 2019). The increase in sympathetic activation in response to hypoxia exposure and its upregulation of intracellular lipolysis has elegantly been demonstrated by Weiszenstein et al. (2016). Specifically, they showed that IH exposure for 14 days in mice significantly increased adipocyte lipolysis and elevated NEFA levels, effects that were attenuated when mice were treated with a pharmacological lipolysis inhibitor, acipimox. In addition to its impact on AT intracellular lipolysis at the whole-body level via the activation of the SNS, hypoxia has also been shown to stimulate basal lipolysis of human subcutaneous (O’Rourke et al., 2013; Mahat et al., 2016, 2018) and visceral (O’Rourke et al., 2013) adipocytes. Cultured adipocytes in hypoxic conditions also showed an activation of the glucose transporter 1, the facilitative glucose transporter independent of insulin, and a reduction in insulin-dependent glucose transporter-4 (Regazzetti et al., 2009; Wood et al., 2011; Varela-Guruceaga et al., 2018). This state of insulin resistance in fat cells is dependent on HIF-1 transcription (Regazzetti et al., 2009; Varela-Guruceaga et al., 2018). Together, these findings provide evidence that hypoxia stimulates intracellular lipolysis and increases the release of NEFA into circulation, which likely explains the marked elevation in circulating NEFA levels commonly observed upon hypoxia exposure (Table 1).

#### Intravascular Lipolysis

Several tissues synthesize the enzyme LPL, the key intravascular lipolytic enzyme. Intracellularly produced LPL is secreted and transported to the luminal side of the capillary endothelium where it hydrolyzes TG contained in circulating TRL (Eckel, 1989; Kersten, 2014). The TG hydrolysis process releases NEFA, which can be taken up by nearby cells. In AT, this cascade results in the intracellular re-esterification of NEFA into storage TG droplets. Therefore, AT-LPL plays a crucial role in the regulation of circulating TG as well as in the AT lipid storage capacity. As summarized in Table 1, rodent studies consistently reported attenuated AT-LPL activity in acute and chronic exposures to hypoxia, where the reduction ranges from 50 to 80% (Drager et al., 2012; Jun et al., 2012, 2013; Yao et al., 2013). In differentiated human preadipocytes, acute hypoxia has been shown to reduce LPL activity dose-dependently (Mahat et al., 2018) with a reduction up to sixfold after 24 h at a FiO_2_ of 0.03 (Mahat et al., 2016). A well-recognized mechanism underlying the reduction of AT-LPL activity upon hypoxia exposure is the upregulation of angiopoietin-like 4 (Drager et al., 2012; Yao et al., 2013), a strong post-translational inhibitor of LPL activity (Wu et al., 2021) induced by HIF-1 (Drager et al., 2013). Together, these findings indicate that hypoxia alters the TG clearing capacity of AT.

### Small Intestine

Ingested fat is the major regulator of CM production in the small intestine (Dash et al., 2015). As previously reviewed (Westerterp and Kayser, 2006; Kayser and Verges, 2013), energy digestibility measured by bomb calorimetry of feces is not affected by altitudes up to 6,500 m, an altitude that refers to a FiO_2_ of ∼ 0.09. Using retinyl palmitate to study the dynamics of buoyant intestinal CM, it has previously been reported that mice exposed to chronic IH show no impairment of intestinal absorption as compared to mice exposed to normoxia (Drager et al., 2012). Recent evidence also suggests that the gastrointestinal tract seems to cope well with hypoxia exposure. More precisely, no difference was observed in the circulating CM-TG of healthy individuals after 6 h of normobaric hypoxia (FiO_2_= 100%) after a meal (Morin et al., 2021).

### Liver

The liver plays a major role in the regulation of TRL, primarily in VLDL production and TRL remnants disposal. VLDL production is modulated by (1) hepatic NEFA delivery, and (2) *de novo* lipogenesis (Nielsen and Karpe, 2012). Also, there is clear evidence that an increase in sympathetic tone to the liver directly leads to an increase in VLDL production (Geerling et al., 2014). As previously mentioned, the increase in sympathetic tone observed upon hypoxia favors both a release of NEFA into circulation, which serve as substrates for VLDL production, and a reduction in TG clearance. It has also been shown that IH, through activation of HIF-1, upregulates a key transcriptional factor involved in lipid biosynthesis, the sterol regulatory element-binding protein-1 (SREPB-1) (Li et al., 2005, 2006). Upregulation of SREPB-1 leads to an increase in the gene expression of several *de novo* lipogenic enzymes, including stearoyl–coenzyme A desaturase 1 (SCD-1) (Shimano, 2001), which facilitates the endogenous synthesis of monounsaturated fatty acids (MUFA). MUFA synthesized through this pathway serve as major substrates for the synthesis of cholesterol esters, TG, and phospholipids (Ntambi and Miyazaki, 2004) that are secreted in the circulation as lipoproteins if said production exceeds the liver’s needs. Overall, hypoxia increases the key substrates involved in VLDL production.

A limited number of studies highlight the impact of hypoxia on the metabolism of TRL remnants. Drager et al. (2012) demonstrated that mice chronically exposed to IH have a decreased clearance of TRL. Interestingly, this effect was also shown in individuals living with severe OSA. Indeed, using radiolabeled lipids, Drager et al. (2018) observed that severe OSA delays both the lipolysis of TRL and the removal of TRL remnants from the circulation. They also demonstrated that the impairments in remnants removal and intravascular lipolysis were strongly correlated with the severity of nocturnal hypoxemia. More recently, a study reported that prandial TG concentrations associated with denser lipoproteins (CM remnants and VLDL) were increased by 6 h of normobaric hypoxia (Mauger et al., 2019). Acute IH was also shown to negatively affect postprandial TG levels in healthy individuals, due to an increase in denser TRL levels such as VLDL and CM remnants (Morin et al., 2021). These results support the concept that hypoxia disturbs the prandial/postprandial metabolism of denser TRL.

## Conclusion

There is ample evidence from murine and human studies linking hypoxia with considerable adverse effects on TG metabolism and triglyceridemia through alterations in AT and liver functions. The present work highlights that hypoxia tends to negatively affect TG levels by increasing the concentration of denser TRL such as VLDL and CM remnants, an observation that mainly occurs in prandial and postprandial states. Taken together, these findings can help to orient future studies or develop strategies to alleviate the effect of hypoxia on TG levels, and therefore reduce the cardiovascular risk associated with hypoxia-inducing health conditions.

## Author Contributions

RM and NG drafted versions of the manuscript with input and revisions from PI. All authors edited, revised, and approved the final version of the manuscript.

## Conflict of Interest

The authors declare that the research was conducted in the absence of any commercial or financial relationships that could be construed as a potential conflict of interest.

## Publisher’s Note

All claims expressed in this article are solely those of the authors and do not necessarily represent those of their affiliated organizations, or those of the publisher, the editors and the reviewers. Any product that may be evaluated in this article, or claim that may be made by its manufacturer, is not guaranteed or endorsed by the publisher.

## Abbreviations

AT: Adipose tissue
ATGL: Adipose triglyceride lipase
CM: Chylomicron
CPAP: Continuous positive airway pressure
CVD: Cardiovascular disease
FiO_2_: Fraction of inspired oxygen
HIF: Hypoxia-inducible factor
HSL: Hormone-sensitive lipase
IH: Intermittent hypoxia
LPL: Lipoprotein lipase
MUFA: Monounsaturated fatty acid
NEFA: Non-esterified fatty acid
O_2_: Oxygen
OSA: Obstructive sleep apnea
SCD-1: Stearoyl–coenzyme A desaturase 1
SNS: Sympathetic nervous system
SREPB-1: Sterol regulatory element-binding protein 1
TG: Triglyceride
TRL: Triglyceride-rich lipoprotein
VLDL: Very-low-density lipoprotein.
